# Atomistic-scale analysis of the deformation and failure of polypropylene composites reinforced by functionalized silica nanoparticles

**DOI:** 10.1038/s41598-021-02460-3

**Published:** 2021-11-29

**Authors:** V. Sorkin, Q. X. Pei, P. Liu, W. Thitsartarn, C. B. He, Y. W. Zhang

**Affiliations:** 1 Institute of High Performance Computing, A*STAR, Singapore, 138632 Singapore; 2 Institute of Materials Research and Engineering, A*STAR, Singapore, 138634 Singapore; 3 Department of Materials Science and Engineering, Faculty of Engineering, National University of Singapore, Singapore, 119077 Singapore

**Keywords:** Theory and computation, Polymers

## Abstract

Interfacial adhesion between polymer matrix and reinforcing silica nanoparticles plays an important role in strengthening polypropylene (PP) composite. To improve the adhesion strength, the surface of silica nanoparticles can be modified by grafted functional molecules. Using atomistic simulations, we examined the effect of functionalization of silica nanoparticles by hexamethyldisilazane (HMDS) and octyltriethoxysilane (OTES) molecules on the deformation and failure of silica-reinforced PP composite. We found that the ultimate tensile strength (UTS) of PP composite functionalized by OTES (28 MPa) is higher than that of HMDS (25 MPa), which is in turn higher than that passivated only by hydrogen (22 MPa). To understand the underlying mechanistic origin, we calculated the adhesive energy and interfacial strength of the interphase region, and found that both the adhesive energy and interfacial strength are the highest for the silica nanoparticles functionalized by OTES molecules, while both are the lowest by hydrogen. The ultimate failure of the polymer composite is initiated by the cavitation in the interphase region with the lowest mass density, and this cavitation failure mode is common for all the examined PP composites, but the cavitation position is dependent on the tail length of the functional molecules. The present work provides interesting insights into the deformation and cavitation failure mechanisms of the silica-reinforced PP composites, and the findings can be used as useful guidelines in selecting chemical agents for surface treatment of silica nanoparticles.

## Introduction

Effective transition from design to production of engineering materials, such as polymer composites, is usually a lengthy and costly process, which requires iterative search for specific microstructures to meet targeted properties subject to various constraints, such as material availability, manufacturability and production cost. The role of computers in materials design has increased markedly over the last several decades mainly driven by the fast development of computing power and more accurate and efficient computational algorithms. Nowadays, computation approaches have been frequently applied to gain the understanding and make predictions of various properties of materials. The study of large-scale phenomena, such as interfaces in polymer composites which involve the polymer matrix, reinforcer and molecular linker, from a bottom-up route requires efficient atomistic modelling techniques, such as molecular dynamics approach. Such simulations will not only allow us to gain the understanding of structure–property relations, but also to select and optimize basic components at the interfaces to improve their properties and to maximize their performance.

Thermoplastic polymers, such as polypropylene, polyethylene and polystyrene et al., have been widely used in the field of packaging, electric and automobile industries due to their low density, high chemical resistance and remarkable thermal stability^1,2^. However, their structural applications are to some extent limited by the low failure strength^3^. The most practical way to enhance the strength of these thermoplastic polymers is to form a composite by uniformly dispersing hard and strong fillers in hosting polymer matrix^4–6^. Considerable research efforts have been dedicated to the optimization of mechanical properties of thermoplastic polymer composites by tuning the shape, size and concentration of their reinforcing fillers^5,7^.

According to previous atomistic simulation studies, once a reinforcing filler is embedded in a polymer matrix, a thin glassy interphase region is formed around it. The thickness of the interphase zone is ~ 1.5–2 nm^8–10^, which is insensitive to the polymer chain length or to the diameter of dispersed nanoparticles^3,9,11–14^. The density of the interphase region is ~ 1.3–2.5 times higher than that of the polymer matrix^15–17^, while its distribution is non-uniform: a few well-defined dense layers appear around the surface due to the attractive polymer-filler interaction^9,10,18–24^. Inside the interphase zone, the polymer may form the sequences of adsorbed segments, extending loops and dangling tails^21,25^. The average gyration radius within the interphase zone is generally larger than that in the bulk^19,26^. The mobility in the interphase region is often significantly reduced due to the polymer crowding and ordering^11–14^.

The presence of nanoparticles surrounded by the interphase regions may considerably improve the mechanical properties of polymer composites^9,10,13,27,28^. Nanoparticles added to polymer matrix can change its mechanical properties in several ways. They can impart additional strength and fracture toughness, inhibit failure by blocking the propagation of cracks, and alter the mechanical properties through the structural changes of polymer layer surrounding the particle surface. Studies based on both atomistic and continuum (micromechanical models) approaches have enabled to gain a better understanding of the mechanisms leading to the improvement of mechanical properties of polymer composites.

Molecular dynamics (MD) simulations have provided the detailed picture of the deformation and failure (at the atomistic scale) of polymer composites reinforced by a variety of fillers, including clay flakes^29,30^, graphene layers^31,32^, carbon nanotubes^33–35^, buckyballs^15^ and silica nanoparticles^13,20,36^. As a result, a number of strengthening mechanisms have been identified.

According to previous MD simulations^37^, a uniaxial tensile strain is able to lead to an affine displacement of chains along the stretching direction in the matrix, but the embedded nanoparticles alter the affine displacement field in the interphase zone. This is an effective strengthening mechanism since an extra stress is required to overcome the non-affinity of polymer displacements while stretching the composite^11,38^. The smaller is the diameter of nanoparticles, the larger is the total volume of interphase region (at fixed weight fraction of filler), and the stronger is the reinforcing effect, as demonstrated by MD simulations of silica/polyethylene^9,39^, silica/polystyrene^11^, buckyball/polyethylene^15^ and CNT/epoxy^40^ nanocomposites. Moreover, the dispersed nanoparticles form a dynamic filler–polymer network^39^ since a polymer chain can adhere to a number of nanoparticles and a nanoparticle can also be in contact with several chains, forming cross-links between them^11^. The temporary cross-links limit the accessible polymer conformations and reduce their mobility, thus effectively strengthening the composites. Since the mobility of nanoparticles is higher than that of polymers, they can rearrange under applied stress to release the local tension and to dissipate the accumulating strain energy, thus further strengthening and toughening the polymers nanocomposites^27,38,39,41^.

Micromechanical models have been developed to study the mechanical properties of polymer composite at the mean field or continuum level. These models are generally of low computational cost and of reasonable accuracy^42^. Among the commonly applied are the rule of mixtures^43^, Kerner^44^ and Lewis-Nielsen^45,46^ models for predicting the stiffness of composites, and the Mori–Tanaka model^47^ for calculating elastic stress field around the embedded particles. According to these models, the nanoparticles included into the polymer matrix give rise to both the weakening and reinforcing effects. The weakening effect arises from the stress concentration around the nanoparticles^48^. The reinforcing effect is due to the load transfer from the matrix to the nanoparticles^49^, provided the adhesion between them is strong. Moreover, the nanoparticles serve as barriers inhibiting crack propagation, which enhances the composite toughness. Several micromechanical models have been recently proposed, which correlate the strength of particulate composites with the adhesion strength, nanoparticles size, their volume fraction and spatial distribution, as well as with the modulus ratio of nanoparticle to polymer matrix^5,48–51^. However, it is still difficult to predict in general the strength for a specific composite due to the complex interplay of these parameters. Moreover, the ultimate failure strength of a particular composite is determined by the path of least resistance for crack which propagates throughout the microstructure, rather than the statistically averaged values of the microstructure parameters^48^.

Previous atomistic simulations indicate that the failure of polymers generally occurs via nucleation and growth of cavities^16,38,52–56^. Such cavities typically start from the non-uniformly distributed free volume regions or voxels with a typical size of a few Å^57^. Under applied tensile strain, these voxels grow, causing their transformation into voids. For polymer composites, such as silica/polybutadiene^55^ and nano-diamond/polymethyl-methacrylate^58^, MD simulations indicate that these voxels are predominantly located near the interphase zone. Under external loading, the voids are preferentially nucleated from these positions, and their subsequent growth and coalescence lead to the loss of loading capacity of the composites^38,53,56^.

A number of micromechanical models have been developed to determine the predominant parameters affecting the fracture in polymer composites^16,39,56,59^. Due to the complexity of the numerous failure and toughening mechanisms operating on multiple time and length scales (including crack pinning, crack-tip blunting, crack deflection by nanoparticles, nanoparticle–matrix interface debonding, cavitation, formation of immobilized polymer interphase around nanoparticles, matrix shear yielding, craze formation and breakage of nanoparticles^6,39,44,45,52,55–59^), up to now, there is no universally accepted micromechanical model of failure^16,39,48,49,56,59–63^.

These detailed understandings of the deformation and failure mechanisms in polymer nanocomposites provide valuable guidelines for the design strategies for their strengthening. Under loading, the applied stress is transferred from the polymer matrix to the fillers through the interphase region. The efficiency of the stress transfer depends on the strength of the interphase region, which is closely related to the nature of the filler–polymer interactions. In general, the stronger the interactions, the more resistant the polymer composite to deformation and failure^35,64^.

One of the most efficient ways to enhance the strength of filler–polymer interactions is the functionalization of nanoparticles, in which special surface agents grafted on the filler surface. Such functionalization can efficiently increase the strength of interphase region since besides the nanoparticles-polymers interaction, there is also interaction between the grafted functional molecules and polymers^65,66^. MD simulations demonstrate that the interphase zone around the functionalized nanoparticle has a layered structure^14,19,26^. The functional molecules can reduce the mobility of polymers in the vicinity of the filler surface and hence have a stronger reinforcing effect^26,36,53,66,67^. The failure strength of polymer composites with functionalized fillers reaches its maximum at a moderate grafting density, which increases in proportion to the interaction strength between polymers and functionalized nanoparticles^53^.

Majority of previous atomistic simulation studies on polymer composites with functionalized fillers were performed using coarse-grained MD (CG-MD) models^19,26,36,53,67^. In CG-MD models, the number of degrees of freedom is significantly reduced and fine interaction details are eliminated^28,68^. In addition, the filler surface was usually functionalized with the same matrix polymers. Hence, despite of the accumulated knowledge on the deformation and failure of polymer composites, the detailed effects of functional molecules on the strengthening of composites are still poorly understood. In contrast to CG–MD simulations, all-atom MD simulations, which include all the atomistic degrees of freedom and atomistic interaction details, are expected to reveal more detailed effects of functional molecules on the strengthening mechanisms of the composites.

Although the PP composites reinforced with silica nanoparticles have been well studied, there are still many open questions and unresolved issues concerning the deformation mechanisms and failure behaviors of these composites. In particular, there is no clear understanding of the failure mechanisms operating at the atomistic level, especially in the case when the silica nanoparticles are functionalized by various surface agents. A clear understanding of the effect of functional agents on the deformation mechanisms and failure modes of PP composites at the atomistic level under different loading conditions can help us to select and design suitable functional molecules for better interfaces, and consequently for better PP composites. In this study, we use all-atom MD simulations to examine the effects of grafted functional molecules commonly used in diverse industrial applications. In particular, we consider a polypropylene as a host polymer matrix, and use silica nanoparticles as a filler. Since silica is an inorganic material, which only weakly interacts with an organic polypropylene matrix, two special functional molecules, namely, hexamethyldisilazane (HMDS) and octyltriethoxysilane (OTES)^26,66,69,70^, are grafted onto the silica surface via covalent bonding. Our objective is to study the effect of surface functionalization of silica nanoparticles by HMDS and OTES molecules on the structure and properties of the interphase region, as well as on the deformation and cavitation failure mechanisms of PP composites subjected to uniaxial tensile load. Our findings can provide valuable insights into the role of functional molecules in enhancing the mechanical properties of polymer composites.

## Computational model

**Figure 1:**
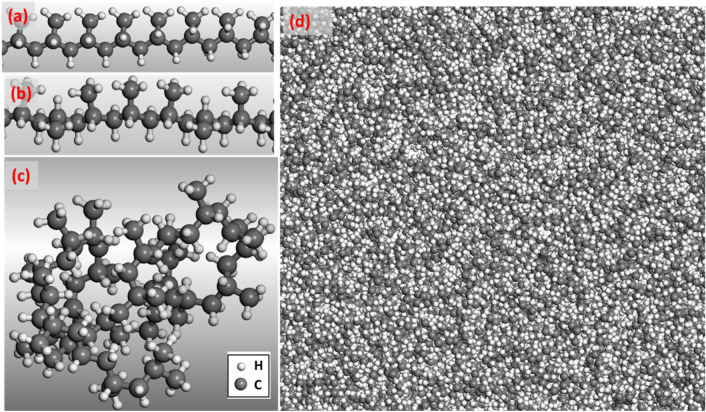
The atomistic model for PP matrix. An isotactic (**a**) and an atactic (**b**) segment of a PP chain. A single PP chain (**c**) taken out of an amorphous PP matrix (**d**). The mass density of the amorphous PP matrix ρ = 0.9 g/cc at room temperature. Carbon atoms are grey and hydrogen atoms are white. The BIOVIA Materials Studio 2020 VERNO 20.1.0.2728 (https://www.3ds.com/products-services/biovia/products/molecular-modeling-simulation/biovia-materials-studio) was used to create the drawings in the figure.

In order to construct a PP composite reinforced with functionalized silica nanoparticles, we initially built the three components individually, that is, polymer matrix, spherical silica nanoparticle and functional molecules, and then assembled them together. We constructed PP polymer chains [CH_3_-CH-CH_2_]_n_ containing n = 120 monomers (each chain consists of N = 209 atoms). A segment of PP backbone is shown in Fig. 1a, in which every second carbon atom has a pendant side methyl group. Typical PP polymer utilized in industrial applications is a mixture of 90% isotactic and 10% atactic segments^2^ (a PP segment is isotactic if all the pendant groups lie on the same side of chain as shown in Fig. 1a; while a PP polymer is atactic if the pendant groups are randomly oriented along the chain as shown in Fig. 1b). Hence, in our MD simulations, we used the isotactic PP polymers with 10% fraction of atactic segments. Figure 1c illustrates one of the various conformations of a single PP chain, and Fig. 1d shows an amorphous PP matrix containing 200 PP chains. The amorphous PP matrix with a mass density of ρ = 0.9 g/cc was built by using Monte-Carlo method as implemented in the ‘Amorphous cell’ module of ‘Materials Studio’^71^.

The effect of microstructure, in particular crystalline lamellae, on the mechanical properties, deformation and failure mechanisms, is an important issue. Experimental observations^72^ indicate that the degree of crystallinity of PP composites changes very little with the addition of silica nanoparticles. This implies a weak interaction of crystallites and reinforcing nanoparticles^72^. The typical size of crystalline domain in neat PP matrix is about ~ 100,000–4,000,000 Å^72^, which is much larger than the typical length scale of MD simulations.

**Figure 2:**
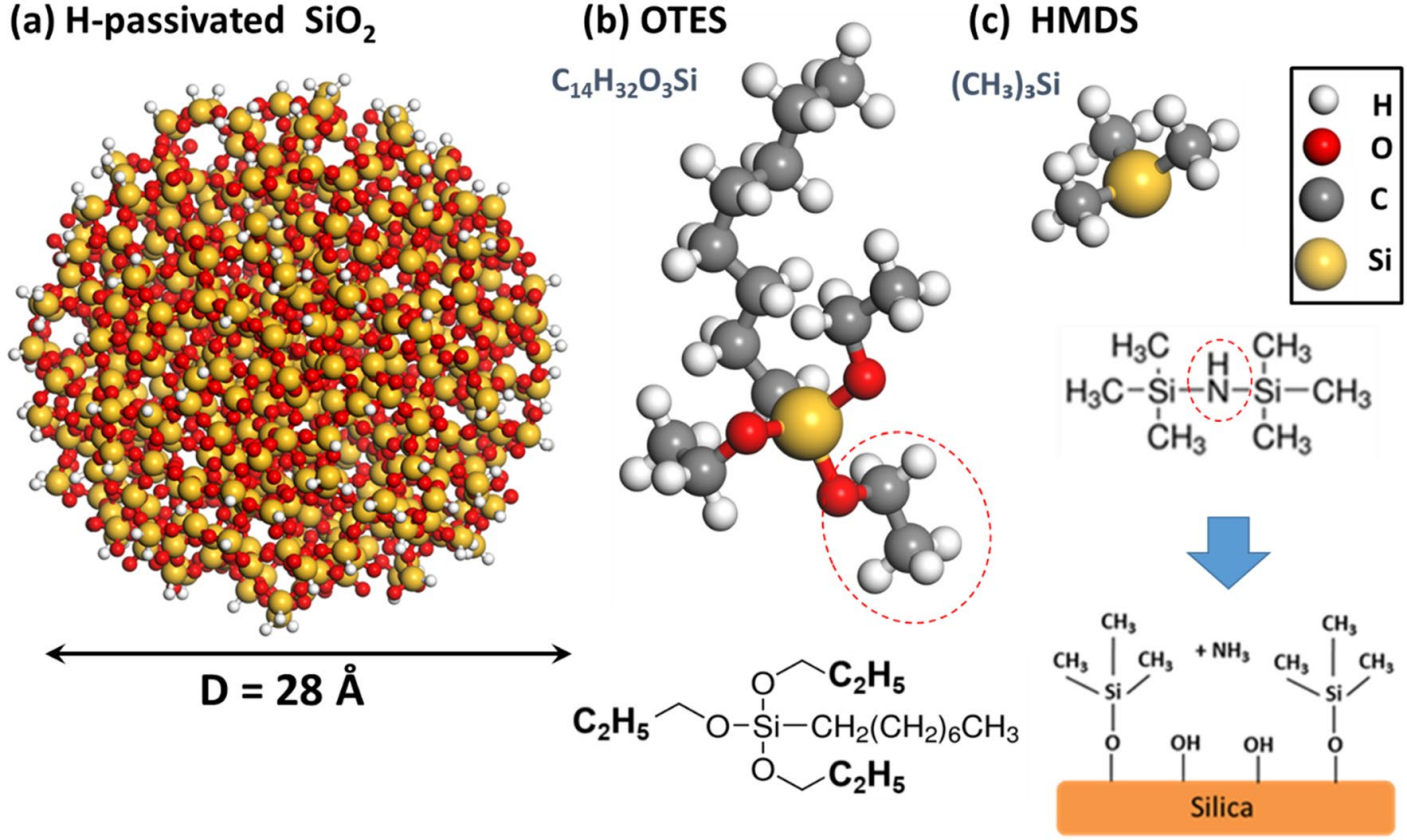
The atomistic model of hydrogen passivated SiO_2_ nanoparticle (**a**) The atomistic models and structural formulas of OTES (**b**) and HMDS (**c**) functional molecules. An ethyl C_2_H_5_ end-group can be released as an ethane C_2_H_6_ molecule during chemical functionalization outlined by red dashed line. During the chemical functionalization, HMDS splits, producing the chemically grafted surface of silica and ammonia^77^. Hydrogen atoms are white, carbon atoms are grey, oxygen atoms are red, and silicon atoms are yellow. The BIOVIA Materials Studio 2020 VERNO 20.1.0.2728 (https://www.3ds.com/products-services/biovia/products/molecular-modeling-simulation/biovia-materials-studio) was used to create the drawings in the figure.

Next, we constructed an atomistic model for silica nanoparticles. A silica nanoparticle with a diameter of D = 28 Å was cut out of an amorphous SiO_2_ bulk generated by ‘Materials Studio’^73^. The selected diameter is sufficiently large to capture the effects of nanoparticle functionalization by surface agents, and reasonably small to make feasible the all-atomistic MD simulations of the mechanical properties of polymer nanocomposite. The surface of the silica particle was modified in the following way: first, we removed all the surface silicon atoms with more than one dangling bond. Second, all the disconnected isolated oxygen atoms were eliminated. Finally, all the remaining silicon atoms with a single dangling bond were passivated by hydrogen atoms (see Fig. 2a). Subsequently, the atomistic configuration of the constructed silica nanoparticle was optimized by the conjugated gradient optimization using COMPASS Force Field^74^.

**Figure 3:**
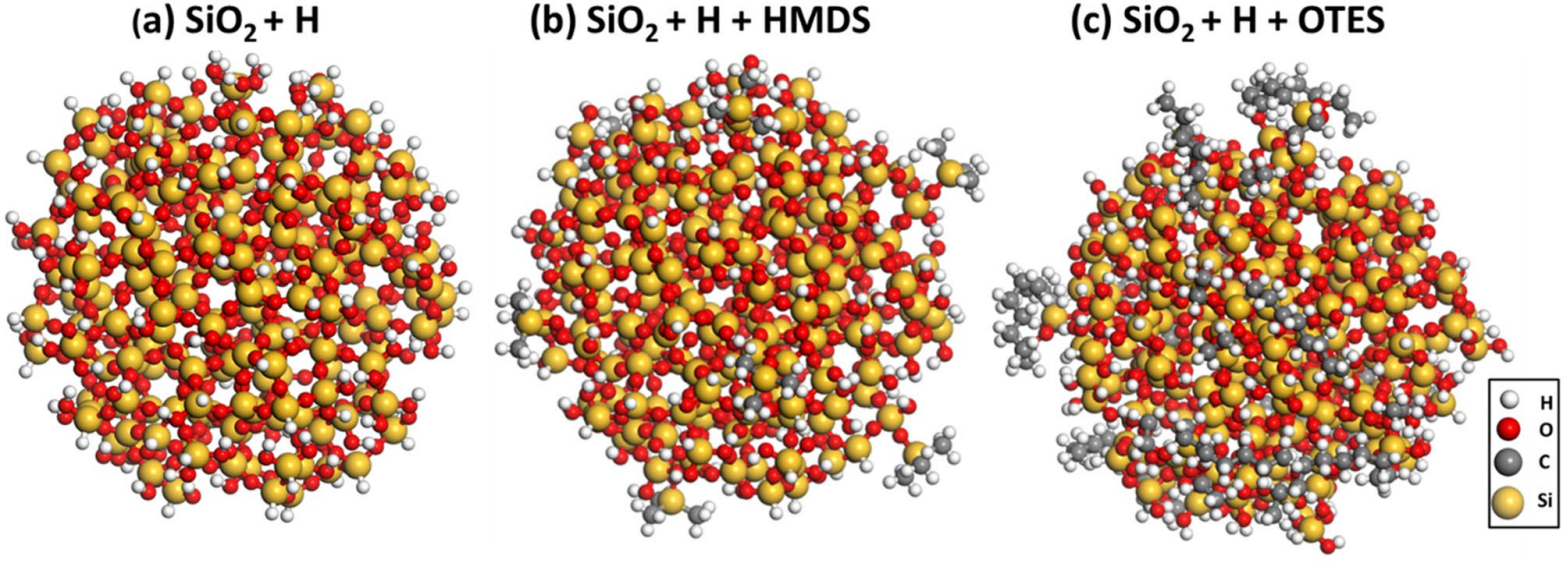
Atomistic models for SiO_2_ nanoparticle with the surface passivated by hydrogen (**a**) and functionalized with HMDS (**b**) and OTES (**c**). The surface coverage with the functional molecules is n_s_ = 5%. Hydrogen atoms are white, carbon atoms are grey, oxygen atoms are red and silicon atoms are yellow. The BIOVIA Materials Studio 2020 VERNO 20.1.0.2728 (https://www.3ds.com/products-services/biovia/products/molecular-modeling-simulation/biovia-materials-studio) was used to create the drawings in the figure.

The atomistic models and structural formulas of the two functional molecules, that is, OTES and HMDS, are shown in Fig. 2b,c, respectively. To begin with, we constructed an OTES molecule, which is a surface agent for functionalization of silica commonly used to improve the mechanical strength of plastic polymers composites^75,76^. The chemical formula of OTES molecule is C_14_H_32_O_3_Si and its molecular weight is M_w_ = 276.49 Da. As can be seen in Fig. 2b, it is an organic silane with three ethyl C_2_H_5_ groups and a relatively long hydrocarbon tail attached to the silicon atom. The ethyl groups often form strong covalent bonds with the surface of silica both in aqueous solution and in air. During hydrolysis reaction, the end groups of OTES attract hydrogen atoms, which passivate the dangling bonds of silicon atoms on the surface, and then transform the C_2_H_5_ ethyl end groups into ethane C_2_H_6_ molecules. Subsequently, the newly formed ethane molecules are released, and the remaining oxygen atoms form covalent bonds with the residual silicon atoms. Typically, one of the oxygen atoms of OTES molecule forms a covalent Si–O bond with a surface Si-atom (see Fig. 2b), although in some cases, two or even three Si–O bonds can be formed^66^. Hence, in the present work, we attached OTES molecules to the surface of silica particle by forming a single covalent Si–O bond, and added a comparatively small fraction (~ 20%) of OTES molecules by forming double or triple covalent Si–O bonds with underlying silica^66^. A typical silica nanoparticle functionalized with OTES molecules is shown in Fig. 3b.

The atomistic model for HMDS functional molecule is shown in Fig. 2c. HMDS is an extremely versatile and strong surface agent, which is commonly used as a modifier to enhance interfacial adhesion of silica particles and to restrict their agglomeration in polymer composites^40^. The chemical formula of HMDS is C_6_H_19_NSi_2_ and its molecular weight is M_w_ = 161.39 Da (see Fig. 2c). HMDS contains two polar functional groups, which play an important role in the coupling between the functionalized silica particles and polymer matrix. In the process surface functionalization, an HMDS molecule splits into two subunits (C_6_H_19_NSi_2_ + 2H → 2((CH_3_)_3_Si) + NH_3_). The residual molecules form strong covalent Si–O bonds with the underlying silica surface as illustrated in Fig. 2c. At the end of the reaction, the N-atom of HMDS molecule is released as a part of ammonia (see Fig. 2c). In our MD simulations, we split HMDS molecule into two (CH_3_)_3_Si subunits and constructed covalent Si–O bonds between them and surface Si-atoms. Therefore, each subunit of the cleaved HMDS molecule is separately covalently bonded to the silica nanoparticles (see Fig. 3c).

The constructed functional molecules of OTES and HMDS were covalently grafted onto the surface of the silica nanoparticle. We set the surface coverage by the functional molecules to $n_{s}$ = 5%, which means that 5% of hydrogen atoms initially passivating the dangling bonds of the surface Si-atoms were replaced by the functional molecules. The corresponding hydrogen atoms were removed and the functional molecules were attached instead by forming covalent Si–O bonds, as described above. In this way, the silica surface was uniformly covered by the functional molecules (see Fig. 3). The designated surface coverage $n_{s}$ = 5% is optimally located in the middle of the range of 1%-10% typically used in most of polymer composites^78^).

In order to embed the functionalized silica nanoparticle into the PP matrix, we used a Monte-Carlo based packing method as implemented in ‘Amorphous cell’ module of ‘Materials studio’^71^. The target packing density of PP polymers was set to ϱ = 0.9 g/cc. In the present study, we constructed three different models of the PP composites: The first one contains the silica nanoparticle only passivated with hydrogen atoms (see Fig. 3a), the second one contains the silica nanoparticle both passivated by hydrogen and functionalized with OTES (see Fig. 3b); and the last one contains the silica nanoparticle both passivated by hydrogen and functionalized with HMDS (see Fig. 3c).

A cubical computational box with the length of $L = 60{\AA}$ was chosen as an initial shape for the supercell of PP composite. The volume fraction of the reinforcing particle is $n_{v} = \frac{{\frac{4}{3}\pi R^{3} }}{{L^{3} }} = 0.05,$ while the weight fraction is about ~ 10%. Periodic boundary conditions were applied along all the three directions. The constructed samples were equilibrated using an NPT ensemble (the number of particles, N, pressure, P, and temperature, T were all kept constant), allowing the volume of computational box and therefore the density to be adjusted. We set the time step to $dt = 0.25 fs$ and applied the Noose-Hover thermostat to control the sample temperature. To speed-up the usually long-time equilibration of polymer composites, our MD simulations started at the elevated temperature of $T = 400K$, which was gradually brought down to room temperature using a series of consequent simulations in the NPT ensemble. After the volume relaxation, the density slightly decreased to $\rho = 0.89 g/cc$, which was close to the target value^79^.

We used COMPASS Force-Field in all the full atomistic MD simulations of the created PP composites since it is considered to be the best ab initio based force-field suitable for a wide range of materials, including PP and silica^74^. After the equilibration, the prepared samples were subjected to uniaxial tensile stress in order to examine the deformation and cavitation failure mechanisms of the PP composites reinforced by the functionalized silica nanoparticles. Uniaxial tensile stress was applied by using the Souza-Martins barostat^80^ within the “Forcite Plus” module as implemented in Materials studio^81^. The Souza-Martins barostat is based on a variable-cell-shape MD algorithm, where the dynamical variables associated with the computational cell are the six independent dot products between the vectors defining the cell. This is the most efficient algorithm capable to simulate the application of anisotropic stress^80^. Our MD simulations were carried out as follows: we applied a uniaxial tensile stress along the X-direction and ran a long MD simulation (for about ~ 50 ns) until the sample was stabilized and equilibrated under the applied tensile load. In this quasi-equilibrium state, we measured the sample strain at a given tensile stress, and examined its molecular structure, in particular, at the interface region in the vicinity of the silica nanoparticle. Then the tensile load was increased again by $\Delta \sigma = 10$ MPa, and the procedure was repeated until the ultimate failure of the sample was reached at a critical tensile stress.

## Results

**Figure 4:**
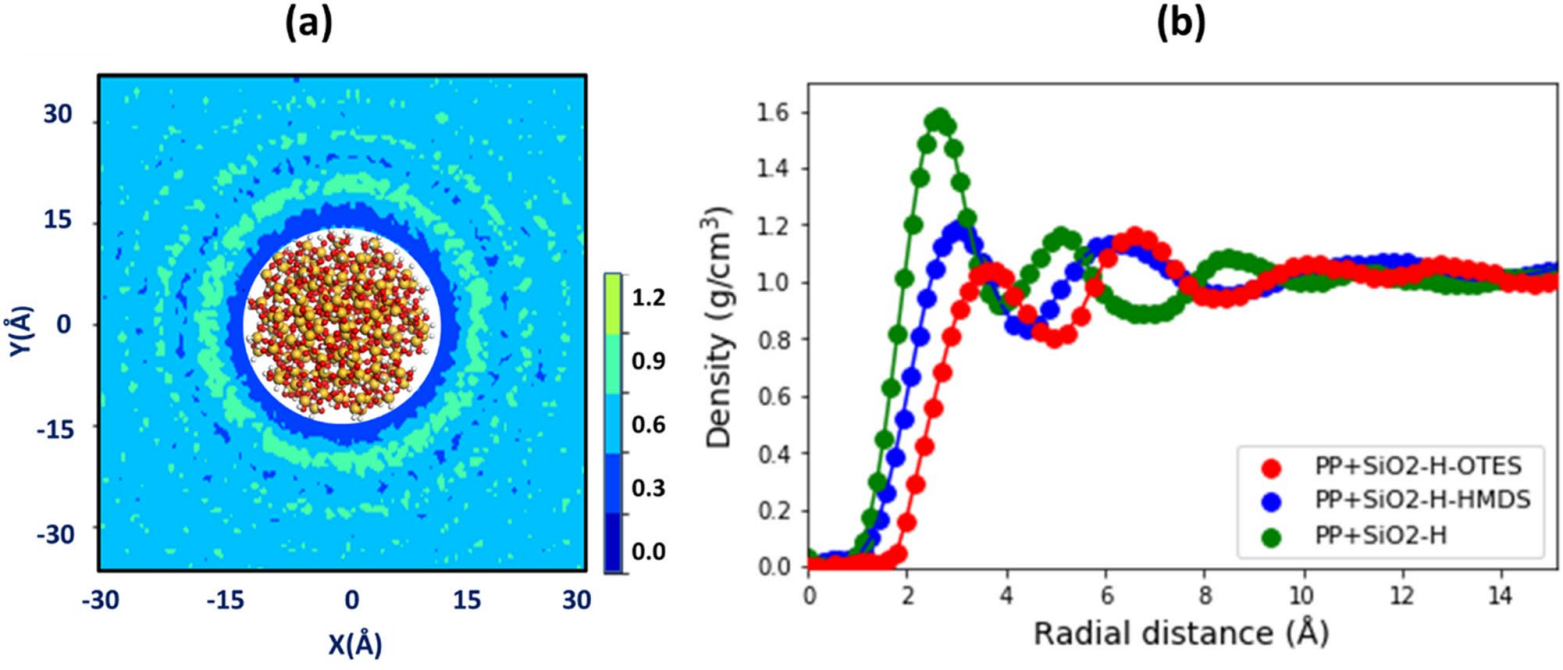
(**a**) The mass density distribution of the PP chains around the SiO_2_ nanoparticle functionalized with the HMDS molecules. The density distribution is obtained in a thick square slab with thickness of ∆ = 5 Å. The color bar indicates the mass density range in units [g/cc]. (**b**) The radial mass density profiles of PP polymers calculated as a function of distance from the surface of functionalized SiO_2_ nanoparticle in the thermal equilibrium. The density profiles of PP for silica particle passivated with hydrogen (red circles) and in addition functionalized with HMDS (green diamonds) or OTES (blue squares) molecules are shown.

To begin, we examined the structure of interphase region formed in the vicinity of the silica nanoparticle in the PP composite in thermal equilibrium at room temperature (see Fig. 4). The most important structural characteristics of interphase is the mass density distribution around the SiO_2_ particle. Figure 4a plots the in-plane polymer density distribution around the SiO_2_ with the grafted HMDS molecules. The density is calculated in a rectangular slab with the thickness of ∆ = 5 Å surrounding the SiO_2_ nanoparticle. The slab is partitioned into a set of small prism domains (0.5 Å × 0.5 Å × 5 Å). The mass density distribution is calculated by counting the mass of atoms in each cell. The density distribution in Fig. 4a is the average over a large set of the PP configurations sampled at thermal equilibrium. As can be seen in Fig. 4a, the interphase region is characterized by the visible density oscillations.

Since the mass density distribution in Fig. 4a can be considered as a spherically symmetrical to a sufficient accuracy, we plot the radial mass density distribution in Fig. 4b by calculating the mass density as a function of distance from the silica surface (see Fig. 4b). In this case, the interphase region surrounding the silica is subdivided into a set of concentric spherical shells. The distance between neighboring shells is set to ∆ = 0.5 Å, while the center of concentric shells is at the center of mass of silica. The radial density profile was obtained for each sampled configuration by calculating the atomic mass of PP polymers within each concentric region and dividing the mass by the volume of that region. The radial density profiles, calculated as an average over all the configurations sampled in equilibrium, are shown in Fig. 4b for the silica nanoparticle passivated by hydrogen (red circles), and in addition functionalized with HMDS (green diamonds) or OTES (blue squares) molecules.

As can be seen in Fig. 4, the specific pattern of the density oscillation appears in all the radial density profiles. This density oscillation is the manifestation of layering^14^ around a spherical nanoparticle, which is similar to that at a planar surface^68,82^. The layering effect is a trade-off between the energy gain, $E$, due to the interaction of polymers leading to their dense close packing at the surface, and the loss of the conformational entropy, $S$, due to this dense packing at the surface, which significantly restricts the conformations accessible to the polymer chains. The emerging density pattern is a realization of the required minimum of free energy ($F = E - TS$) for thermodynamic equilibrium^14^ at a given temperature, *T*. The layered structure is characterized by the distinct density oscillations near the surface, while at a certain distance from the surface, the amplitude of density oscillations diminishes and converges rapidly to the average bulk value of propylene matrix (see Fig. 4b). The distance at which the density converges to the average value of polymer matrix is used to estimate the size of interphase region. As can be seen in Fig. 4b, the thickness of the interphase ($t \approx 10\;{\AA}$) region is nearly the same for the PP composites containing silica nanoparticles functionalized with the different molecules.

We also found that regardless to the type of functional molecules grafted on the surface, the average size of the polymer chains, measured by the squared radius of gyration, is larger in the interphase region than in the polymer matrix (the difference ~ 10%), and the chain segments are preferentially orientated along the silica surface. The grafted HMDS molecules leave enough space for polymer chains to approach close to the surface, while OTES molecules, with the longer hydrocarbon tails partially, efficiently exclude the polymer chains from the immediate vicinity of the surface, as can be seen in the radial density profiles of PP polymers in Fig. 4b.

**Figure 5:**
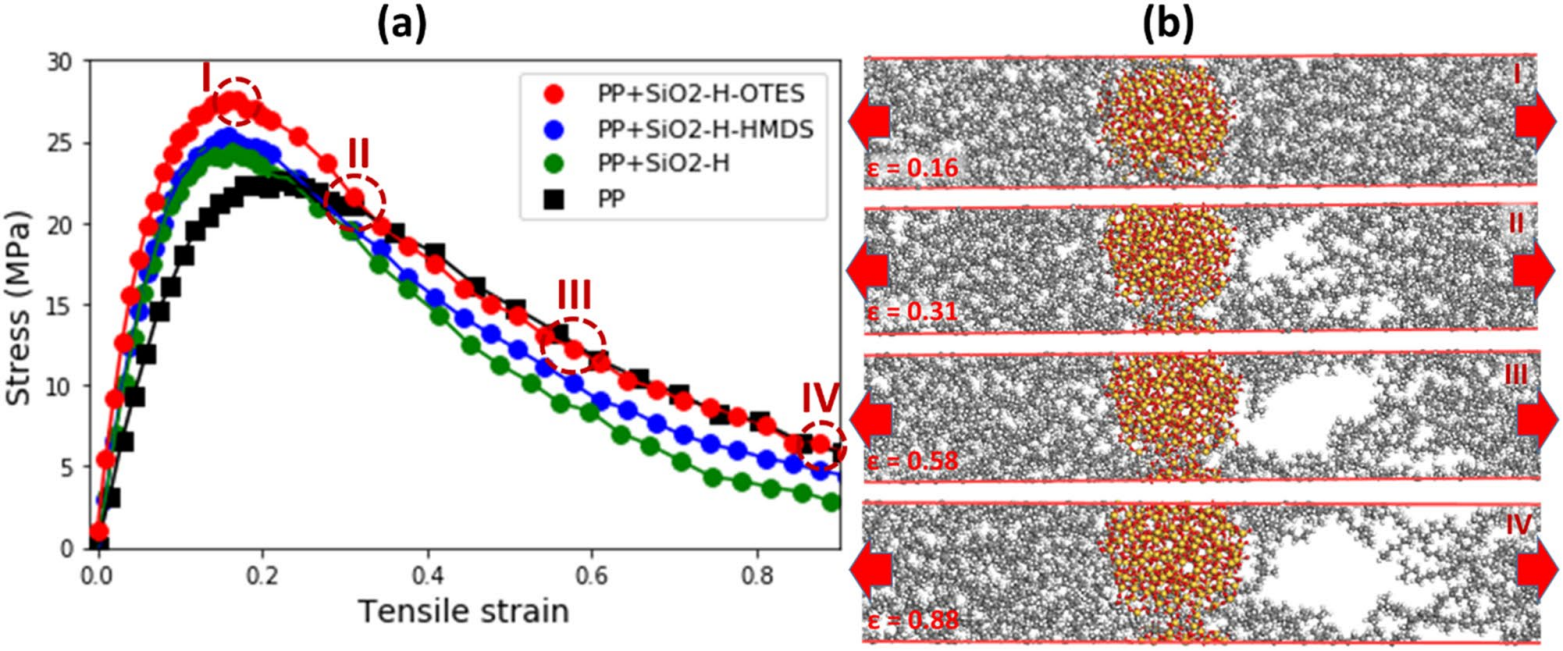
(**a**) Stress–strain curves calculated from the deformation of the PP composites subjected to an applied uniaxial tensile stress. The PP composites containing silica nanoparticles passivated only by hydrogen (green circles) and in addition functionalized with HMDS (blue circles) or OTES (red circles) molecules are shown. The stress–strain curve for the amorphous PP matrix (black squares) is added for comparison. The sequence of atomistic snapshots corresponding to the selected points (I, II, III and IV) on the stress–strain curve for the PP composite with SiO_2_ passivated by hydrogen and functionalized with OTES is shown in (**b**). The mechanism of failure of PP composite via cavitation. The failure occurs via the void formation and growth in the vicinity of functionalized SiO_2_ nanoparticle. The nucleated void grows rapidly, leading to the ultimate failure. Red arrows indicate the tension direction.

After the examination of the interphase region formed around the functionalized silica nanoparticle, we subjected the PP composites to a uniform uniaxial tensile stress. The samples were quasi-statically stretched along the X-direction. Using the equilibrated samples as the reference configurations, we calculated the tensile strain at a given stress. The obtained stress–strain curves for the PP composites containing SiO_2_ passivated by hydrogen (green circles) and in addition functionalized with HMDS (blue circles) or OTES (red circles) molecules are plotted in Fig. 5a, while the stress–strain curve for the pure PP matrix (black squares) is added for comparison.

Using the obtained stress-strength curve, we calculated the Young’s modulus for neat PP matrix and PP composites. The acquired values are compared with those predicted by a few selected micromechanical models. First, we calculated the upper and lower limits of Young’s modulus for the PP composite reinforced by silica nanoparticles using a micromechanical model based on the rule-of-mixtures (ROM)^43^ model. The upper bound, ${\text{E}}_{{\text{c}}}^{{\text{u}}}$, calculated for the parallel arrangement of two-phases (matrix and filler), is given by^43^: ${\text{E}}_{{\text{c}}}^{{\text{u}}} = {\text{E}}_{{\text{m}}} \left( {1 - {{\upvarphi }}_{{\text{p}}} } \right) + {\text{E}}_{{\text{p}}} {{\upvarphi }}_{{\text{p}}} =$ 4.4 GPa, where ${{\upvarphi }}_{{\text{p}}}$ is the volume fraction of SiO_2_ nanoparticles (${{\upvarphi }}_{{\text{p}}} = 0.05$), while ${\text{E}}_{{\text{m}}}$ and ${\text{E}}_{{\text{p}}}$ are the Young’s moduli of neat PP matrix^83^ (${\text{E}}_{{\text{m}}}$ = 0.95 GPa) and silica^84^ (${\text{E}}_{{\text{p}}} { }$ = 70 GPa), respectively. The lower limit for the Young’s modulus of PP composite, ${\text{E}}_{{\text{c}}}^{{\text{l}}},$ obtained for the case of serial arrangement of two-phases, is given by^43^: ${\text{E}}_{{\text{c}}}^{{\text{l}}} = \frac{{{\text{E}}_{{\text{m}}} {\text{E}}_{{\text{p}}} { }}}{{{\text{E}}_{{\text{m}}} {{\upvarphi }}_{{\text{p}}} + {\text{E}}_{{\text{p}}} (1 - {{\upvarphi }}_{{\text{p}}} )}} = 0.{99}$ GPa.

The application of one of most versatile equations for polymer composites with spherical particles proposed by Kerner^44^ gives: ${\text{E}}_{{\text{c}}} = {\text{E}}_{{\text{m}}} \left( {1 + \frac{{{{\upvarphi }}_{{\text{p}}} }}{{1 - {{\upvarphi }}_{{\text{p}}} }}\frac{{15\left( {1 - {\upnu }_{{\text{m}}} } \right)}}{{8 - 10{\upnu }_{{\text{m}}} }}} \right) = 1.07\;{\text{GPa}}$, where ${\upnu }_{{\text{m}}}$ is the Poisson’s ratio of PP matrix (${\upnu }_{{\text{m}}} = 0.43)$^1,83^. The obtained value lies between the upper and lower limits given by the ROM model. According to the generalized equation for the elastic moduli of composite materials suggested by Lewis-Nielsen^45,46^: ${\text{E}}_{{\text{c}}} =$ ${\text{E}}_{{\text{m}}} \left( {\frac{{1 + {\text{AB}}\varphi _{{\text{p}}} }}{{1 - {\text{B}}\varphi _{{\text{p}}} }}} \right) = 1.16$ GPa, where ${\text{B}} = \frac{{\frac{{{\text{E}}_{{\text{p}}} }}{{{\text{E}}_{{\text{m}}} }} - 1}}{{\frac{{{\text{E}}_{{\text{p}}} }}{{{\text{E}}_{{\text{m}}} }} + {\text{A}}}}$ and A = 2.47 is the parameter that takes into account the particle size and Poisson's ratio of the PP matrix^45,46^. Finally, as stated by the model proposed by Counto^85^, in which perfect bonding between the filler particles and the polymer matrix is assumed, the Young’s modulus is given by: ${\text{ E}}_{{\text{c}}} = \left( {\frac{{1 - \sqrt {{{\upvarphi }}_{{\text{p}}} } }}{{{\text{E}}_{{\text{m}}} }} + \frac{1}{{\frac{{1 - \sqrt {{{\upvarphi }}_{{\text{p}}} } }}{{\sqrt {{{\upvarphi }}_{{\text{p}}} } }}{\text{E}}_{{\text{m}}} + {\text{E}}_{{\text{p}}} }}} \right)^{ - 1} =$ 1.29 GPa. All the results predicted by these micromechanical models are comparable with the experimental measurements obtained by Wu et al.^83^: ${\text{E}}_{{\text{c}}} =$ 1.24 GPa at the volume fraction of silica nanoparticles ${{\upvarphi }}_{{\text{p}}} = 0.0{3}$ in isotactic semi-crystalline polypropylene PP matrix (with degree of crystallinity of  ~ 50%). In comparison with neat PP matrix, the inclusion of SiO_2_ nanoparticles increases ${\text{E}}_{{\text{c}}}$ by ~ 30%.

A variety of parameters (adhesion strength, particle size, volume fraction and spatial distribution) tend to change the elastic modulus in a non-linear fashion^86^. Consensus has not been reached yet on the primary reason of why nanocomposites exhibit a larger increase in the Young’s modulus compared to microcomposites. The most plausible hypothesis is that nanoparticles form ultra-large interfacial area at the same volume fraction of filler. An increase in the elastic modulus is generally observed with reduction of nanoparticle size (provided that the nanoparticle–matrix adhesion is strong)^87^. This is particularly not clear, as the Young’s modulus is almost independent of the interfacial adhesion: the modulus is measured at relatively low tensile deformation, hence there is insufficient deformation to cause interface separation, thus the adhesion strength should not noticeably affect the Young’s modulus. However, if adhesion is strong, then interphase can be formed around the nanoparticles with intermediate elastic properties between those of the nanoparticles and matrix. When the interphase was taken in the micromechanical models^87^ into account, it was found that this vast interfacial region can remarkably increase Young’s modulus.

In our MD simulations, the value of Young’s modulus was calculated by using the slope of stress–strain curve for PP composites at relatively small tensile deformation. We found that the Young’s modulus for PP amorphous matrix$,{\text{ E}}_{{\text{m}}} =$ 0.62 GPa, is smaller than that of semi-crystalline PP. However, the inclusion of silica nanoparticles increases the Young’s modulus by ~ 25% (up to ${\text{E}}_{{\text{c}}} =$ 0.78 GPa), which is consistent with the increase in elastic module observed experimentally and predicted by the micromechanical models.

**Figure 6:**
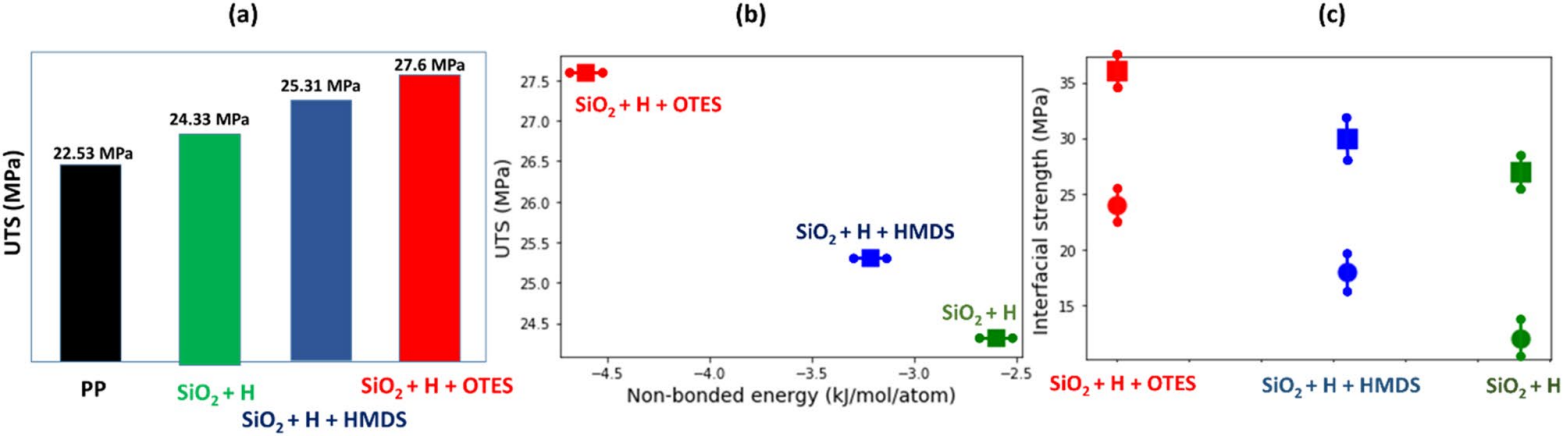
(**a**) The UTS of PP matrix (black) as compared with the PP composite containing the silica nanoparticles passivated by hydrogen atoms (green) and in addition functionalized with HMDS (blue) or OTES (red) molecules. (**b**) The UTS vs. non-bonded interaction interphase energy for the PP composites with silica nanoparticles passivated by hydrogen atoms (green squares) and in addition functionalized with HMDS (blue squares) or OTES (red squares). (**c**) Interfacial strength of PP/silica calculated for tensile (squares) and shear (circles) loading modes^66^. A planar silica surface is passivated by hydrogen atoms (green) and in addition functionalized with HMDS (blue) or OTES (red) molecules. Error bars are indicated for the non-bonded interaction interphase energy and interfacial strength in (**b**,**c**).

It is evident that introduction of the reinforcing silica nanoparticles strengthens the PP composites (see also Fig. 6a). Second, the functionalization of SiO_2_ surface further increases the strength of PP composites as compared to the non-functionalized silica nanoparticles. Third, there is a noticeable difference in the effect of different grafted functional molecules on the failure stress of PP composites, as can be seen in Fig. 5a. The effect of OTES molecules is stronger than that of HMDS molecules.

In order to compare the strengthening effect, we calculated the ultimate tensile stress (UTS) defined as the maximal tensile stress that the polymer composite is able to bear before final failure, $\sigma_{f}$ (see Figs. 5a, 6a). We found that the UTS for pristine PP matrix is $\sigma_{f} =$ 22.53 MPa. When the SiO_2_ nanoparticles passivated by hydrogen atoms were used to reinforce the polymer matrix, the UTS of the composite increases to $\sigma_{f} =$ 24.33 MPa (an increase of ~ 8%). If 5% of the hydrogen atoms passivating the silica surface are replaced by the grafted HMDS molecules, the UTS increases further up to $\sigma_{f} =$ 25.31 MPA (an increase of $\sim$ 12% as compared to the PP matrix). However, the strongest effect was found when OTES molecules were grafted on the surface instead of HMDS ones, which increased the UTS even further to $\sigma_{f}$ = 27.36 MPa (an increase of $\sim 21$% in comparison to the PP matrix).

The observed increase in tensile strength of PP composite by silica nanoparticles is in agreement with micromechanical models predicting strengthening of polymer matrix by filler, provided a strong particle–matrix adhesion is ensured^87^. The accurate prediction of the strength of composites with micromechanical models is difficult as it is determined by the complex interplay of the different parameters such as adhesion strength, stress concentration, size, volume fraction and spatial distribution of the filler.

In order to explain the strengthening effect, Bigg^50^ proposed a model in which the composite strength, ${\upsigma }_{{\text{c}}}$, is given by: ${\upsigma }_{{\text{c}}} = {\upsigma }_{{\text{m}}} \left( {1 - {\text{a }}\varphi_{{\text{p}}}^{{\text{b}}} + {\text{c }}\varphi_{{\text{p}}}^{{\text{d}}} } \right)$, where ${\upsigma }_{{\text{m}}}$ is the polymer matrix strength, ${{\upvarphi }}_{{\text{p}}}$ is the filler volume fraction, {a, b, c, d} is a set of empirical constants. In this model, the term-$\upsigma _{{\text{m}}} {\text{a}}\varphi _{{\text{p}}}^{{\text{b}}}$ describes the weakening effect due to the stress concentration induced by the nanoparticles, while the term $\upsigma _{{\text{m}}} {\text{c}}\varphi _{{\text{p}}}^{{\text{d}}}$ describes the reinforcing effect due to the interfacial adhesion and load transfer from the matrix to the nanoparticles. Turcsanyi et al.^51^ generalized this model for the case of very strong particle–matrix interfacial bonding:$\upsigma _{{\text{c}}} = \upsigma _{{\text{m}}} \left( {\frac{{1 - \upvarphi _{{\text{p}}} }}{{1 - 2.5\upvarphi _{{\text{p}}} }}} \right){\text{e}}^{{{\text{B}}\upvarphi _{{\text{p}}} }}$, where B is an empirical constant, which is determined by the surface area of particles, particle volume fraction and interfacial bonding energy.

There have been a few attempts to correlate the strength of particulate composites with the mechanical properties of interphase layer formed around the hosted nanoparticles^48,88–91^ Voros et al.^88^ developed a micromechanical model of polymer composites with nanoparticles surrounded by interphase layer. The layer stiffness is the highest at the particle surface and gradually decreases to the matrix value. The formation of the stiff interphase layer leads to a decrease in the local stress concentration and an increase in the local yield stress around the nanoparticles. As a result, the probability of interfacial debonding decreases and yielding does not occur near the particle surface, but in the polymer matrix^88^. Dubnikova et al.^89^ developed a micromechanical model of the interfacial debonding in PP composites, and identified a critical volume fraction of filler at which an uncorrelated particle debonding is transformed into a correlated one. The effect of correlated particle debonding is helpful for understanding the strengthening of PP composites by silica nanoparticles.

Kim and Michler^90,91^ proposed a micromechanical model with ‘three-stage’ mechanism of deformation and failure of polymer composites based on interfacial debonding. According to the model, nanoparticles act as stress concentrators, altering the stress state in the surrounding polymer matrix^63,91,92^. The stress concentration increases proportionally to applied tensile stress. The stress concentration causes a volume dilatation, which results in void formation in the surrounding matrix. The maximum stress concentration is at the poles of nanoparticles where the debonding is initiated. The cavitation ultimately induces extensive plastic deformation in the polymer matrix, such as shear yielding and multiple crazing^90,91^. It is evident that our simulation results are consistent with the Kim and Michler^90,91^ model predictions.

A direct comparison between the results of our MD simulations and of the micromechanical models is difficult without the knowledge of empirical constants for the specific PP composite with silica nanoparticles.

As the applied tensile stress was gradually increased, a critical stress was reached at which the strained PP composite started to yield and ultimately failed. Figure 5b illustrates the cavitation failure process of the PP composite reinforced by silica nanoparticles passivated by hydrogen atoms and functionalized with OTES molecules. As can be seen in Fig. 5b, the cavitation failure process was initiated after a void with a radius of $R\sim 5 {\AA}$ appeared in the vicinity of the silica surface within the interphase region. In order to identify the distance from the silica surface to the site of void nucleation, we used the ‘Density Field’ feature of the ‘Forcite Plus’ module as implemented in the ‘Materials Studio’ software^73^ to map the density of the sample to a smoothed three-dimensional density field. Next, we created a three-dimensional isosurface (by setting it at 10% of the maximum density value) to identify the surface of the nucleated void. We started our analysis with the well-defined void (as shown in Fig. 5b at the tensile strain of ε = 0.31), and then rolled backward by tracing frame-by-frame the void shrinkage to the minimal size. To a good approximation, this small void was considered as a sphere, and the center of which was considered to be the void nucleation site. We found that the void nucleation occurred in the vicinity of the lowest mass density position in the interphase region. Not surprisingly, the position of the lowest mass density and the void nucleation site are different for polymer composites with different functional molecules due to the difference in the size of the functional molecules. In particular, the largest distance from the silica surface $r_{n} \cong 5.0$ Å was observed for the silica nanoparticles functionalized by OTES molecules with a long hydrocarbon tail, while the distance of $r_{n} \cong 4.4$ Å was observed for the smaller HDMS molecules, and the smallest distance of $r_{n} \cong 3.9$ Å for hydrogen atoms.

The void nucleation was associated with the separation between the functionalized silica and the surrounding PP polymers. Since the sample was continuously stretched along the X-direction, the void grew rapidly, ultimately leading to the cavitation in the PP composite. A sequence of the atomistic snapshots taken prior to the failure via cavitation is shown in Fig. 6. For the cases, we found that the cavitation failure mode is typical for all the PP composites containing silica particles. At the critical stress, a void nucleated in the interphase zone almost at the same distance and subsequently grew rapidly. A separation between the PP matrix and the functionalized SiO_2_ nanoparticles initiates at atomistic scale the ultimate failure of the composite.

In our MD simulations, cavitation occurs in the polymer matrix rather than in the immediate vicinity of the silica surface. This is in agreement with the predictions based on the micromechanical model developed by Chen et al.^49^. By using the Eshelby’s equivalent inclusion method, they calculated the stress difference just before and after the debonding for a single particle embedded in a polymer matrix under uniaxial tensile loading. Using an energetic analysis, they defined a critical particle radius below which the cavitation occurs in the polymer matrix surrounding the nanoparticle. For the range of interfacial strength obtained in our MD simulations, the value of critical radius is about R = 50 Å, which is larger than the radius of SiO_2_ (R = 12 Å) in the studied PP composite.

## Discussion

In this section, we discuss the effect of functionalization on the deformation, failure via cavitation and strengthening of PP composites reinforced by silica nanoparticles. First, we consider the effect of functionalization on the deformation mechanism. Under an applied uniaxial tensile stress, the polymer chains of the PP composites are continuously reoriented and elongated along the stretching direction. Yet, within the interphase region, the attractive polymer–filler interaction constrains these conformational changes. The restriction of accessible polymer conformations, and the corresponding reduction of their mobility are effectively ways to strengthen polymer composites. The strongest confining effect arises from the OTES molecules due to their long hydrocarbon tails, which are able to strongly interact with the polymer chains. The influence of the HMDS molecules is comparatively weaker due to their short hydrocarbon tails. As a result, a significantly larger tensile stress is required to deform the PP composites reinforced by the silica nanoparticles functionalized with OTES (see Fig. 5a).

The tensile stretching of the PP composites leads to the cavitation at a critical tensile stress at atomistic level. We found that in all the examined PP composites a void nucleates in the vicinity of the silica at a distance of $r_{n} \sim 8$ Å at a site with the comparatively low mass density, and continuously grows because of the applied uniaxial tensile loading. In order to understand why the nucleation occurs near the interphase region, we estimated the average strength of the non-bonded polymer–polymer and polymer-nanoparticle interaction, and found that the latter is weaker than the former, and thus the void nucleation starts in the interphase region as the polymer chains can be relatively easily separated from the functionalized filler in the interphase zone under the tensile stretching. The void nucleates only if the applied tensile stress is strong enough to overcome the polymer-filler attraction. The functionalization of silica surface enhances the attractive non-bonded polymer-filler interaction, and therefore, a noticeably larger tensile stress is required to induce the cavitation near a functionalized silica surface. In particular, we noticed that the polymer-filler interaction strength is considerably enhanced by functionalization of the silica surface with OTES molecules. The impact of HMDS molecules on the interaction strength is relatively weaker although it is still noticeable as compared to surface passivation only by hydrogen atoms. We also observed that the functional molecules must be grafted within an optimal range of surface coverage: if the surface coverage is too low the strengthening effect is too weak, but if the coverage is too high, the mutual interaction of the functional molecules likewise reduces the strengthening effect.

In order to demonstrate that the functionalization of the silica surface enhances the strength of the polymer–filler interaction (and as a result increases the UTS of the PP composites), we estimated the adhesion energy between the PP matrix and the embedded functionalized silica nanoparticle. The adhesion energy is calculated as the energy of all non-bonded interactions including both van der Waals and Coulomb interactions^93^. The non-bonded energy of the interphase region represents the total pair-wise energy between the functionalized silica nanoparticle and PP polymers located within a cut-off radius ($R_{c}$ = 10 Å). With the aim to calculate the non-bonded energy of the interphase zone, we constructed an atomic set containing all the atoms of the functionalized silica nanoparticle, including the hydrogen atoms and atoms of the grafted functional molecules. For each atom in the constructed set, we identified all its neighbors that: (1) belong to the PP chains and (2) located within the range of $R_{c}$ ≤ 10 Å from the given atom. If an atom is not surrounded by the polymers with the selected cutoff distance, it is excluded from the constructed atomic set. By using the COMPASS Force Field, the pair-wise non-bonded energy was obtained for every atom in the atomic set. The adhesion energy was calculated as a sum of the non-bonded energies over all atoms in the constructed set. In order to compare the effect of different functional molecules, the obtained adhesion energy is normalized by the number of atoms in the constructed atomic set (see Fig. 6b). As can be seen in Fig. 6b the adhesion energy per atom is the largest if the surface of silica functionalized with OTES molecules ($E_{ad} = - 4.8$ kJ/mole/atom) and the smallest the surface of silica is passivated by only the hydrogen atoms ($E_{ad} = - 2.6$ kJ/mole/atom)$.$

An alternative way to quantify the interfacial strength is to examine the mechanical behavior of the PP/silica composite under the tensile and shear loading by using a planar functionalized surface and a polymer layer^32,33,66^. Using full atomistic MD simulations, Pei et al.^66^ calculated the interfacial strength between a PP layer and a planar silica surface passivated by hydrogen atoms and, in addition, functionalized with HMDS molecules. Using the same approach, we calculated the interfacial strength for a planar silica surface functionalized with OTES molecules (see Fig. 6c). As can be seen in Fig. 6c, the maximal interfacial strength, in both tensile and shear modes, is obtained when the silica surface is functionalized with OTES molecules, and the minimal interfacial strength is obtained for the silica surface passivated only by hydrogen atoms. These results are consistent with those obtained by using the non-bonded energy of the interphase region (see Fig. 6b,c), confirming that the functionalization with OTES molecules substantially increases the polymer-filler interaction strength. In addition, we note that similar to the void nucleation case near the surface of the embedded spherical silica nanoparticles, the detachment of the PP polymer layer in tensile deformation mode begins at the functionalized surface of silica.

The obtained values for the tensile strength of PP composite reinforced with silica nanoparticles are in a good agreement with experimentally measured ones. For example, Reynaud et al.^94^ measured up to ~ 10% increase in the tensile strength for SiO_2_ nanoparticles with the average diameter of D = 100 Å at filler loading of ${\text{w}}_{{\text{f}}}$ = 6 wt%; Kim et al.^88^ measured ~ 12% and Wu et al.^83^ ~ 10% increase in the tensile strength for the similar PP composites. In addition, Rong et al.^95^ also found that the tensile strength of PP composite increased by ~ 15% when SiO_2_ particles were functionalized by polystyrene. Hence, these increases measured in previous experiments are consistent with our simulation predictions.

Next, we compare our full atomistic results with those obtained by CG-MD approach. Since we are unable to find CG-MD simulations for PP composites reinforced with silica nanoparticles functionalized by OTES and HMDS molecules, our comparison is here rather indirect and largely qualitative. It is known that in using the CG-MD model, one often represents an entire functional group of atoms by a single bead, thus substantially reducing the number of degrees of freedom to be simulated. In addition, rather than using a complex force field, the beads interact often through a simple short range pair-wise potential, which approximates the essential chemistry of the system. Therefore, the major advantage of a CG-MD model is its ability to access considerably longer length and time scales, however, under the expense of accuracy^10,24,28^. Qualitatively, both the full atomistic and CG-MD methods are able to predict the same trends for some of the features, for example, layering, chain expansion and preferential orientation of polymer segments along the nanoparticle surface, as well as hindering of elongation and reorientation of chains by nanoparticles under tensile deformation, and void nucleation prior to failure^11,14,28,37,96,97^. However, a full atomistic approach is able to take into account the fine details of atomic and molecular configurations of polymer composites. For example, in all-atom MD simulations, the magnitude of mass density variation is noticeably smaller than that obtained for similar composites by CG-MD simulations^10,11,14,24,28,37,96,97^. Also in an all-atom model, the silica nanoparticle surface is rough at the atomistic scale, and some segments of the PP chains can access the surface regions that are not shielded by the functional molecules, while the nanoparticle is often modelled as an ideal smooth sphere in CG-MD models. Last but not least, due to the significant reduction in the degrees of freedom, molecular chains become significantly more rigid in CG-MD model, which is unable to capture accurately the position and initial stage of void nucleation from voxels at atomistic level. It is expected that the detailed information on the deformation and failure mechanisms of PP composites reinforced by functionalized silica nanoparticles revealed by the present all-atom MD simulations provides a valuable reference for both future CG-MD simulations and experimental studies.

Finally, we briefly discuss the effect of polymer length on the mechanical properties of PP composites. It is well-known that an increase in polymer length (molecular weight) of PP chains leads to the formation of long ligaments (fibrils)^90,91,98^ crossing and connecting the opposite sides of voids appearing in the polymer matrix during cavitation. According to the experiment^98^, the ligament strength increases proportionally to the length (or molecular weight) of PP chains. The stronger ligaments may affect the deformation and failure of PP composites by inhibiting the void growth during cavitation. In addition, provided that nanoparticle–polymer interaction is sufficiently strong, an increase in polymer length may lead to a stronger adsorption of polymer chains on the surface of the nanoparticles, which in turn promotes a better dispersion of nanoparticles^99^ in the polymer matrix. Moreover, an increase in the polymer length may also enhance both the impact toughness of PP composites (since the inherent ductility of the neat PP matrix increases with the molecular-weight of PP chains^100^) and the tensile strength (since the degree of chain alignment depends on the molecular weight^101^). On this basis, it is likely that the deformation and failure mechanisms of PP composites reinforced with SiO_2_ nanoparticles can be affected by the length (molecular weight) of PP chains. Due to the limited length scale reachable by our full atomistic MD simulations, the length of PP polymer was fixed at a specific value. An important future work will be to study the effect of PP length on the deformation and failure mechanisms of PP composites.

## Conclusions

Using full atomistic MD simulations, we investigated the effect of surface functionalization of reinforcing silica nanoparticles on the deformation and cavitation failure mechanisms of PP composites. In the present work, the surface of silica nanoparticles was passivated by hydrogen atoms and in addition functionalized with HMDS or OTES molecules. First, we examined the interphase region formed around the silica nanoparticles embedded in the PP matrix. The oscillated polymer density distribution around the functionalized nanoparticles was investigated and a number of densely packed layers were identified. The change in orientation of the backbone segments of chains results in the efficient packing in the interphase region. The size of interphase region is only weakly affected by the type of functional molecules. Next, we investigated the effect of filler functionalization on the deformation and failure via cavitation at atomistic scale of the PP composites subjected to a uniform uniaxial tensile load. We found that functionalization of the silica surface enhances the strength of polymer-filler interaction, and therefore, strengthens the PP composites. Grafting enhances the interaction strength since besides the polymer-filler interaction, there is also interaction between the functional molecules and polymers. An increase in the strength of attractive filler–polymer interaction induced by the functional molecules determines the extent to which PP composites can be strengthened. The strengthening effect is larger if the silica surface functionalized with OTES rather than with HMDS molecules. To measure the increase in the strength of attractive filler–polymer interaction induced by the functional molecules, we calculated the adhesive (non-bonded) energy of the interphase region and its interfacial strength. Both the adhesive energy and interfacial strength are found to be higher when OTES molecules are grafted on the silica surface, while surface passivation by hydrogen is least effective. More specifically, the UTS for the sample with the silica nanoparticles functionalized with OTES molecules is $\sigma_{f} = 28$ MPa, which is higher than $\sigma_{f} = 25$ MPa for the case with HDMS molecules grafted on the silica surface, which is in turn higher than $\sigma_{f} = 22$ MPa for the case with only hydrogen passivation. The cavitation failure is determined by the strength of adhesion in the interphase region, which can be improved by functionalization. The ultimate failure of the polymer composites is initiated by cavitation in the interphase region at a critical tensile stress. The nucleated voids grow rapidly, eventually leading to the cavitation failure of PP composites. The failure through the cavitation is found to be common for all the examined PP composites. Our work reveals a number of interesting molecular level insights into the silica-polypropylene coupling in PP composites, and provides useful guidelines for the selection of functional molecules.
